# Numerical Simulation of an Oil Mist Particle Emission and Gas–Oil Separation Device of a Closed Machine Tool in the Post-Environmental Area

**DOI:** 10.3390/ijerph192416415

**Published:** 2022-12-07

**Authors:** Xin Wang, Yuhong Liu, Jinchi Zhao, Yujing Zhou, Fei Wang

**Affiliations:** School of Environment and Architecture, University of Shanghai for Science and Technology, Shanghai 200093, China

**Keywords:** sealing machine, oil mist particles, escape and emission, gas–water–oil mist separation

## Abstract

Owing to the airflow field within airtight machines, oil mist particles escape with the airflow from the machine shell gaps and are emitted externally to the post-environmental area, causing air pollution and threatening workers’ health. The existing local exhaust system is ineffective in capturing oil mist particles. This study proposes a gas–oil separation device that can “in-situ control” the oil mist particles in situ and weaken their outgoing emission and that uses numerical simulations to compare and analyse the emission characteristics of oil mist particles, before and after the addition of the separation device at different exhaust air volumes and particle emission speeds, and to design the structural parameters of the device to improve the separation efficiency of oil mist particles. The structural parameters of the proposed device are designed to improve the separation efficiency of oil mist particles. Studies have shown that for every 200 m^3^/h increase in exhaust air volume, the capture efficiency increases by around 3%, and the particle concentration at the gap in the machine loading door decreases from 9.4 × 10^−7^ kg/m^3^ to 7.7 × 10^−7^ kg/m^3^. The overall escape rate of oil mist particles is in the range of 10–13% after the addition of a pressure relief device. Numerical simulations are performed to analyse the effects of inlet airflow velocity, folding plate spacing, and folding plate angle on the separation efficiency of oil mist particles. Results show that an increase in the inlet velocity of the airflow increases the particle separation efficiency. The most suitable structural parameters for the separation device and the machine are as follows: 60° angle of the folding plate and 30 mm distance between plates, where the separation efficiency is above 80%, and the average separation efficiency is about 86%. The results of this study can be used as a reference for the study of the emission of oil mist particles from enclosed mechanical cutting machines.

## 1. Introduction

China is the world’s largest manufacturing country [1]. With the high pollution-emitting characteristics of many factories, oil mist particle pollution is widespread. For the metal-cutting machine-tool oil mist concentration measurement method [2], the PM10 maximum concentration value should not be greater than 5 mg/m^3^. The “Recommended Standard: Occupational Exposure to Metalworking Fluids” [3] published by the American Institute for Occupational Safety and Health recommends an exposure concentration limit of 0.4 mg/m^3^ (respirable particles) or 0.5 mg/m^3^ (total particles).

**Figure 1:**
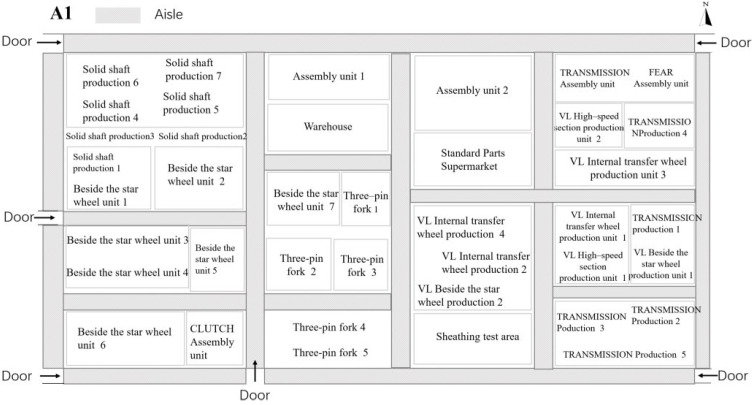
A1 distribution map of the workshop production unit.

To understand the current situation of the factory environment, this study measured the concentration and particle size of oil mist particles suspended in the air in the environmental area of a closed machine tool post in a machining process in Shanghai. The workshop is a large comprehensive machining plant with production cells divided into solid shafts, outer star wheels, three-pin forks, VL inner star wheels, VL outer star wheels, VL high speed joints, VLMONOB, and various assembly cells, as shown in Figure 1. Each production cell consists of a varying number of production lines equipped with machine tools and equipment placed mostly in two parallel rows, with aisles in between for the passage of the operators. The machining processes on each line are mainly grinding and extrusion, medium frequency hardening, tempering, turning, cleaning, and thread cutting. The production process in each line is fully automated, with some of the machines being transported to and from the machining stations by transport belts after machining has been completed. Some of the machines are coupled with robotic arms or workers, so that when the workpiece is finished, the machine loading door opens automatically, and the robotic arm or worker performs the workpiece changeover operation and starts the next round of machining. The workshop is equipped with a closed machine enclosure and a local exhaust system for each of the machining machine points involved in the test.

**Figure 2:**
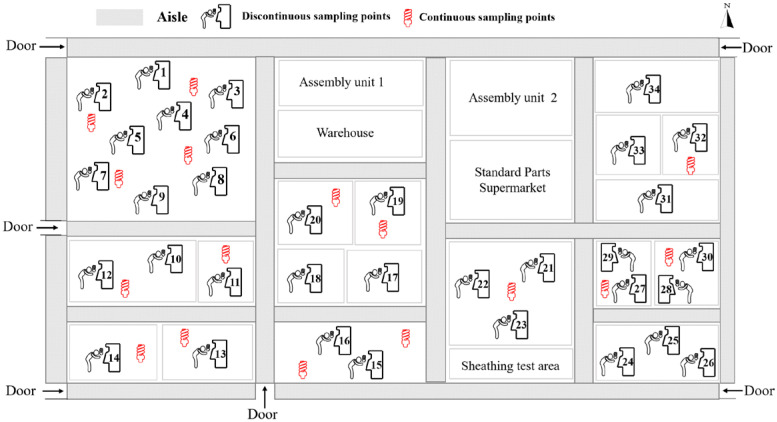
Measurement point layout.

This study used the production unit as the division benchmark, and the different processing equipment of each production unit was selected as the test point. The oil mist concentration measurement was performed approximately 20 cm from the machine tool equipment in the post environmental area. Separate fixed continuous concentration monitoring and mobile discontinuous measurements are conducted in the environmental area of the machine tool post. This study specifies a measurement point at a height of 1.5 m above the breathing zone of the personnel. The measurement time of the fixed continuous monitoring point is 10 min, which needs to ensure that the production cycle of the machine tool is greater than or equal to three times during each sampling period, allowing the particle counter to measure the instantaneous emission concentration of the machine tool for the full working cycle several times, thereby reducing the measurement variability. During the stationary continuous test, the machine tool process is simultaneously recorded on camera to facilitate subsequent analysis of particle emission patterns. The mobile discontinuous measurement has a sampling time of 1 min, and each measurement point is cycled twice, thereby reducing the average concentration difference. The measurement points are arranged on the basis of the actual situation in the workshop, as shown in Figure 2. The test instrument (LIGHTHOUSE particle counters, USA, Handheld3016IAQ) is a LIGHTHOUSE particle counter from the USA.

The average mass concentration of oil mist particles at different machining processes in the machine tool post environmental area was analysed by aggregating data from mobile measurement points. Approximately 54% of the machine tool particle emitting concentrations exceeded or were close to the standard limit of 0.5 mg/m^3^ at the measurement points. The actual measurements and analyses indicate that the oil mist mass concentration is higher than the standard limit of 0.5 Â mg/m^3^ for more than 50% of the sampling points in all the tested process equipment. The average total polar material (TPM) mass concentration of the wet-cutting process with a much higher concentration of oil mist particle emission is approximately 0.53 mg/m^3^, while the average TPM mass concentration of the heat treatment process is approximately 0.63 mg/m^3^. The concentration of oil mist in the machining workshop exceeds the standard, which can significantly harm health [4,5,6,7,8,9].

Therefore, much research has been conducted on oil mist particle emission characteristics and control strategies for different machining processes. Dasch et al. [10,11] studied a large amount of oil mist generated in high-speed grinding machines owing to the collision between the grinding oil and high-speed grinding wheels. The results show that the generation mechanism of oil mist can be divided into centrifugal and evaporation condensation. The former generates particles with diameters concentrated around 3–5 μm, and the latter eventually condenses into small droplets centred at 0.2 μm. Dasch et al. [12] further conducted a qualitative comparison of the factors affecting oil mist generation such as machine cutting speed, tool diameter, and cut depth. They found that the cutting speed has the greatest effect on oil mist concentration and particle size distribution. Khettabi et al. [13] compared the magnitude of oil mist mass concentration in an experimental machining chamber for the minimum flow lubrication mode cutting and dry cutting machining. The experimental results prove that the MQL mode using the cutting fluid produces more oil mist particles and increases particle emission with increasing flow rate. A higher cutting speed also increases the particle size. Wang et al. [14,15] used the Rosin–Rammler function to fit the oil mist particle size distribution law during cutting and established a model of oil mist particle emissivity for each particle size under the dumping mechanism. Although the mechanism, concentration range, and particle size distribution of oil mist particles in machining shops have been extensively investigated, different states of motion are generated in accordance with the internal flow field characteristics of the machine tool when oil mist particles are generated inside a closed machine tool. However, studying the state of motion in actual machine tool machining engineering is difficult because the oil mist particles are ultrafine particles.

Numerical simulations have been used to study particle motion dispersion based on the discrete phase model (DPM), where the particle term is solved by tracking the trajectory of a large number of particles, bubbles, and droplets, combined with a continuous term to solve for the particle motion dispersion state. Wang et al. [16] used the DPM model to study the motion of droplets in an open tank in a push-pull flow field. They concluded that the droplet motion is related to the initial droplet diameter and airflow field. Yang et al. [17] numerically investigated the motion of a single sulphuric acid droplet during free fall using the DPM model and found that a 100 μm sulphuric acid droplet, which first decreases in diameter and then maintains a constant equilibrium diameter, can continue to fall in still air until it reaches the surface. In comparison, a 10 μm droplet takes more time. Duan et al. [18,19] adopted a numerical model to study the dispersion patterns of transiently generated high-temperature particles in industrial processes. With different particle sizes and temperature factors, the model describes the dispersion of particles in the vertical direction based on three indicators: the particle dispersion radius, rise distance, and movement time. Zhuang et al. [20] analysed the dynamics of particle forces through numerical simulations to reveal the mechanism of particle motion. The results showed that the conversion between thermal and kinetic energies drives the upward two-phase flow. The gravitational force acting on the particles and the vortex interaction of the airflow lead to different particle motion behaviour in the vertical and horizontal directions. The above studies listed more factors affecting the diffusion motion of oil mist particles in a closed machining machine. However, the lack of research on oil mist particle emissive motion inside closed machine tools has led to a lack of efficiency in controlling it. Therefore, this study uses a computational fluid dynamics-DPM to study the escape of oil mist particles from a closed machine tool. We then discuss the changes in the flow field inside the machine tool to further explore the causes of oil mist particle dispersion and escape.

Further research on machining shops reveals that current machining shops are dominated by closed CNC machine tools. Most factories add local ventilation systems to machine tools [21,22,23,24,25] to control the oil mist particles generated inside machine tools during machining. Therefore, many scholars have proposed optimisation schemes for local exhaust systems to achieve higher pollutant particle removal efficiency. Cao et al. [22] equipped a vulcaniser with a single local exhaust hood owing to the random release of pollutants during rubber vulcanisation. They experimentally and numerically demonstrated that rubber fumes can be reliably captured when the exhaust air volume is 4000 m^3^/h. Yang et al. [26] used numerical simulations to study the motion of droplets under two types of local ventilations: upper and push–pull exhaust ventilations. They analysed the differences in the control effect of various initial diameters and exhaust velocities on particle capture removal. Lim et al. [27] set up different shapes of exhaust hood dividers to investigate the effective exclusion of pollutants. Chern et al. [28] investigated the addition of exhaust hood baffles and found that the addition of baffles could block the lateral airflow and thus improve the capture efficiency. Zhao et al. [29] optimised the rotating jet of the Aaberg exhaust hood and used a cyclonic field to change the propagation characteristics of pollutants, thus improving the capture efficiency of the exhaust system. Wang and Cao et al. [23,30,31,32] created air supply effects to rapidly capture and eliminate pollutants by changing the air supply angle. The results show that the discharge efficiency of cyclone ventilation is higher than that of the downward air supply and upward air exhaust. In addition, the capture efficiency of transiently released pollutants was higher. However, the duct- and purification device resistance significantly reduce the pumping control efficiency of the local exhaust system for oil mist particles. Therefore, this study attempts to increase the air–water oil mist separation device with pressure relief on two sides of the machine tool, guiding the internal airflow field of the machine tool to carry the oil mist particle flow to two sides of the pressure relief device diffusion and reducing the probability of oil mist particles escaping from the machine tool gap to the outside. The proposed device can be directly used at the source of the machine tool to achieve in situ control and efficient separation and purification, reducing the machine tool post environmental area outside the escape of oil mist particles.

In summary, this study selects an actual closed machining machine as the study object for field measurements. The particle capture efficiency and escape rate of oil mist particles are analysed through numerical simulation under different exhaust air volumes, particle velocities, and particle sizes, and the reasons for the escape of oil mist particles are investigated. A gas–oil separation device consisting of a W-shaped hooked folding plate and high-efficiency filter material with pressure relief is proposed, and the critical inlet velocity value of the separation device and its optimal structural parameters are analysed. The particle separation efficiency of the air–water–oil mist separation device is characterized with regard to different influencing factors, such as inlet air velocity, folding plate spacing, and folding plate angle. This process results in an “in situ control” model for the self-purification of oil mist particles in closed machine tools.

## 2. Analysis of the Formation Process of Oil Mist Particles Escaping from a Closed Machine Tool

### 2.1. Numerical Simulation and Its Physical Model

**Figure 3:**
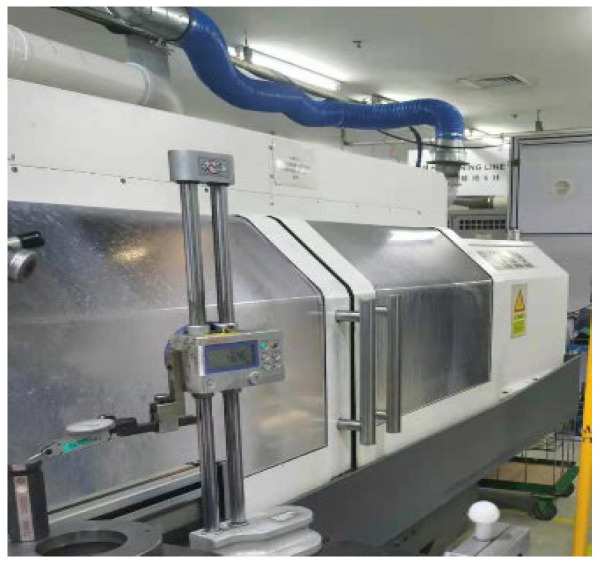
Actual shape of the enclosed mechanical cutting machine.

**Figure 4:**
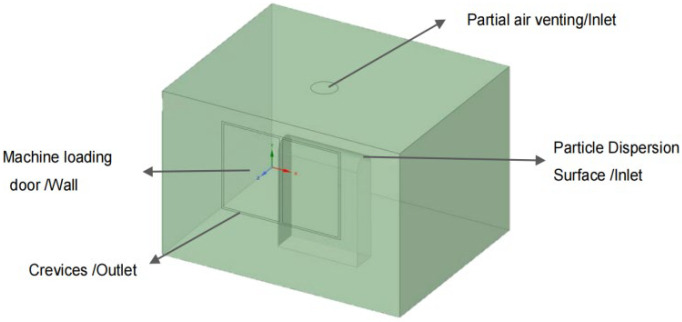
Physical model.

Figure 3 shows the prototype of the mechanical cutting machine; the top section of the particle-emitting source of this machine is a local air vent with a height of approximately 1 m from the processing part. The calculation domain of the physical model has a dimension of 3 m × 2.4 m × 2.25 m, as shown in Figure 4. The overall width of the machine-loading door was 1.2 m, and the height was 1.5 m. The gaps around the loading door of the machine were 0.025 m wide. The simplified oil mist particle emitting source is a semi-circular surface with 0.15 m diameter and 1.3 m from the ground. The local exhaust outlet is simplified as a circle with a radius of 0.3 m, and the rest of the structure is simplified as a wall surface.

#### 2.1.1. Calculation Models for Temperature and Airflow Fields

In the simulation, the fluid in the computational domain is considered to be incompressible, the ambient temperature in the computational domain is set to 27 °C, and the gravitational acceleration is set to gy = −9.81 m/s^2^ and is in accordance with Boussinesq’s assumptions [33,34,35,36]. The structure of temperature and velocity in the computational domain conforms to the continuity equation, momentum equation, and energy equation [37], as shown in Equations (1)–(3):

(1) $$\frac{\partial \rho }{\partial t}+u\cdot \nabla \rho =0,$$

(2) $$\rho \frac{\mathrm{Du}}{\mathrm{Dt}}=-\nabla p+\rho g+\mu \nabla^{2},$$

(3) $$\rho \frac{D\left[\frac{1}{2}\left(u^{2}+v^{2}+w^{2}\right)\right]}{\mathrm{Dt}}=-u\cdot \mathrm{grad}\;p+u\left(\frac{\partial \tau_{\mathrm{xx}}}{\partial x}+\frac{\partial \tau_{\mathrm{yx}}}{\partial y}+\frac{\partial \tau_{\mathrm{zx}}}{\partial z}\right),\;+v\left(\frac{\partial \tau_{\mathrm{xy}}}{\partial x}+\frac{\partial \tau_{\mathrm{yy}}}{\partial y}+\frac{\partial \tau_{\mathrm{zy}}}{\partial z}\right)+w\left(\frac{\partial \tau_{\mathrm{xz}}}{\partial x}+\frac{\partial \tau_{\mathrm{yz}}}{\partial y}+\frac{\partial \tau_{\mathrm{zz}}}{\partial z}\right)+u\cdot S_{M}$$

In this study, Ansys Fluent 2021R1 software is used to solve the temperature, velocity, and particle concentration fields. The pressure parameters are discretized by using the body-force-weighted method. The remaining parameters, such as momentum and energy, are discretized in second-order windward format, the coupled pressure–velocity operation is performed by using the SIMPLE algorithm [38,39], and the Realizable k–ε model is chosen for the turbulence model.

#### 2.1.2. Oil Mist Particle Calculation Model

The DPM is used in this study to investigate the efficiency of the local exhaust air outlet in trapping oil mist particles and the escape rate of particles at the gap of the machine loading door. The model continuous phase takes the calculated results of the Eulerian approach and the trajectory of the discrete phase in the flow field, and its induced heat mass transfer in Lagrangian coordinates is simulated. The liquid phase is considered a continuum by solving the Navier–Stokes system of equations, and the particle phase is solved by tracking a large number of particles, granules or bubbles in the fluid field. Therefore, the two-phase flow field is simulated by using the DPM, where the turbulent diffusion is enabled by the Lagrangian random walk model (DRW).

The basic idea of the Lagrangian method is to calculate the trajectory of the particles and then convert the trajectory to the concentration of the particles. The control equation is based on Newton’s law of conservation of momentum [40] and is shown in Equation (4):

(4) $$\frac{d{\vec{u}}_{p}}{\mathrm{dt}}={\vec{F}}_{d}+\vec{g}\left(\frac{\rho_{p}-\rho_{a}}{\rho_{a}}\right)+{\vec{F}}_{x},$$

where ${\vec{u}}_{p}$—Velocity vector of particles in the air; ${\vec{F}}_{d}$—The drag force vector received by a particle in the air; $\rho_{p},{\;\rho }_{a}$—The density of particles and the density of air; $\vec{g}$—Acceleration of gravity; ${\vec{F}}_{x}$—Other additional forces.

When the Reynolds number is small (Re < 1), the particle is considered to be in the Stokes region, and the drag force is calculated as shown in Equation (5):

(5) $${\vec{F}}_{d}={\vec{F}}_{D}\left({\vec{u}}_{a}-{\vec{u}}_{p}\right)=\frac{18\mu }{\rho_{p}d_{p}^{2}C_{c}}\left({\vec{u}}_{a}-{\vec{u}}_{p}\right),$$

where μ—Air dynamic viscosity; $d_{p}$—Particle diameter; $C_{c}$—Cunningham correction factor is $1+\frac{2\lambda }{d_{p}}\left(1.257+0.4e^{-\left(\frac{1.1d_{p}}{2\lambda }\right)}\right)$.

The main additional forces acting on the particles are Basset force, pressure gradient force, virtual mass force, Brownian force, thermophoretic force, and Saffman lift force. In accordance with the analysis of the literature, the drag force and gravity have the largest order of magnitude, whereas the rest of the forces are of smaller order of magnitude [41]. This study considers Brownian diffusion and Saffman lift forces in addition to gravity and drag forces because the object used is oil mist particles with a small particle size distribution.

For turbulent flow, this study uses the Reynolds-averaged Navier–Stokes model to decompose the airflow velocity around the particles, including the average term and the random term. The random term is represented in the random walk model (DRW), which conforms to a Gaussian probability distribution, as shown in Equation (6):

(6) $${\vec{\;u^{\prime }}}_{a}=\zeta \sqrt{{{\vec{\bar{\;u^{\prime }}}}_{a}}^{2}}=\zeta \sqrt{\frac{2k}{3}}$$

where k—Turbulent power energy; ζ—Random distribution coefficient.

After calculating the particle trajectory, the DRW model needs to be combined with the PSI-C algorithm [42] to count the particle retention time in each grid, which translates into a particle concentration value, as shown in Equation (7):

(7) $${\bar{C}}_{j}=\frac{\;\dot{M}\sum_{i=1}^{n}{\mathrm{dt}}_{\left(i,j\right)}}{V_{j}}$$

where ${\bar{C}}_{j}$—Particle concentration of the jth grid; $\dot{M}$—Number of particles per particle track flow; $V_{j}$—Volume of the jth grid; ${\mathrm{dt}}_{\left(i,j\right)}$—The time taken by the i trajectories to move in the jth grid.

The above calculation method suffers from a high degree of randomness in the calculation process, so the number of tracking particles needs to be increased to eliminate random errors. Yang et al. found that the simulation results are better when the number of particles released from the source was greater than 10,000 by comparing the simulated data and experimental data [43]. This study reduces random errors in particle concentration calculations by encrypting the emitting source grid and individual grid particle releases to a total of 29,382 tracked particles.

In this study, a two-way coupling approach is used to consider the interaction of discrete phase trajectories and continuous phase flow. The number of oil mist particles trapped by the exhaust air outlet and the number of particles escaping from the gap in the machine loading door can be obtained by tracing the particle trajectory to calculate the trapping efficiency and escape rate.

### 2.2. Mesh Independence Studies

The correctness of the numerical simulation results is linked to the number of meshes. A small number of grids causes an increase in the error of the simulation results, but excessive grids reduce the efficiency of the simulation. Therefore, this study considers that the number of meshes can meet the requirements of the calculation when the simulation results no longer change remarkably with the increase in the number of meshes.

**Figure 5:**
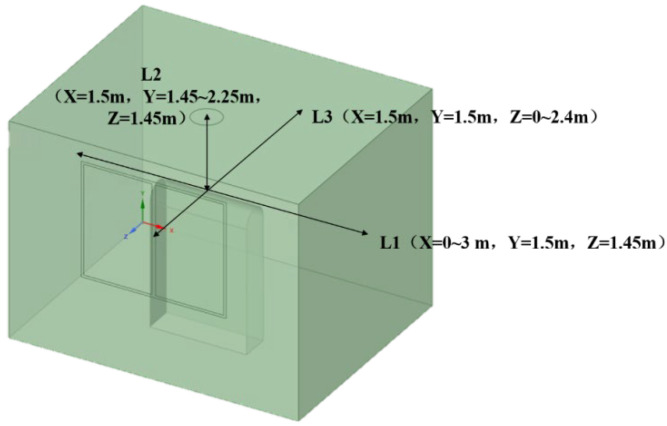
Variation of speed for different number of grids.

In this study, SpaceClaim was used to model and generate the mesh by DM. As shown in Figure 5, three measurement lines L1 (X = 0–3 m, Y = 1.5 m, Z = 1.45 m), L2 (X = 1.5 m, Y = 1.45–2.25 m, Z = 1.45 m), and L3 (X = 1.5 m, Y = 1.5 m, Z = 0–2.4 m) were selected, and their velocity variables were used as the basis for the mesh independence test.

**Figure 6:**
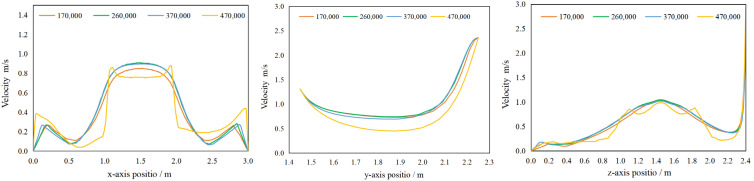
Variation of speed for different number of grids.

The velocity variation of each measurement line for a local exhaust airflow of 600 m^3^/h and a particle velocity of 1.31 m/s at the source of particle emission, with a grid size of 170,000, 260,000, 370,000, and 470,000, are shown in Figure 6.

As shown in Figure 6, the wind speed on the L1, L2 and L3 measurement lines does not vary greatly with the increase in grid number when the grid number is 260,000–370,000. The wind speed has a tendency to be small under the grid number of 170,000, whereas the wind speed fluctuates more obviously under the grid number of 470,000. Therefore, the simulation results at 260,000 and 370,000 grids are not related to the number of grids, and the number of grids used in this model is 260,000, considering the quality of the grids and the length of the simulation.

### 2.3. Numerical Simulation Conditions

In this study, we investigate the influence of two parameters, namely, the exhaust air volume and the particle emission velocity, on the oil mist particle capture efficiency and fugitive rate. Thus, we set up 11 simulated working conditions at four exhaust air volumes for three initial particle emission velocities, as shown in Table 1.

### 2.4. Analysis of Simulation Results

#### 2.4.1. Simulation Validation

Considering that the simulation model is based on a simplified version of an actual machining machine, the simulation boundary conditions are set in accordance with the actual parameters and then compared with the measured data to verify the usability of the simulation model. 

A closed computer numerical control (CNC) cutting machine based on the simulation model in the workshop is selected as the actual measurement object. Considering the instability of machining occasions to exclude various machining processes, worker operations, and other interference factors, the measurement time is set to 2 min, and the number of measurements is set to three times for the fixed continuous measurement of oil mist particle concentration in the machine tool post environment area. The average concentration is used to calculate the average emissivity of oil mist particles by using an average emissivity of 7.17 × 10^−7^ kg/s as the validation data, as shown in Table 2.

Simulations are conducted to verify the parameters set in condition 4. Here, the particle emissions at the source are set to 1 × 10^−6^, 1.5 × 10^−6^, and 2 × 10^−6^ kg/s. The simulated data for the three conditions are compared with the measured data, and the results are shown in Table 3.

According to Table 2, when the particle emission at the emitting source is set to 1.5 × 10^−6^ kg/s, the relative error between the simulated and measured data is the smallest. Thus, this particle emission rate was selected as the boundary condition for the subsequent emissions of the source simulation. This result shows that the model calculation results are accurate and can be used for the subsequent conclusion analysis.

#### 2.4.2. Velocity Field and Particle Concentration Field Analysis

**Figure 7:**
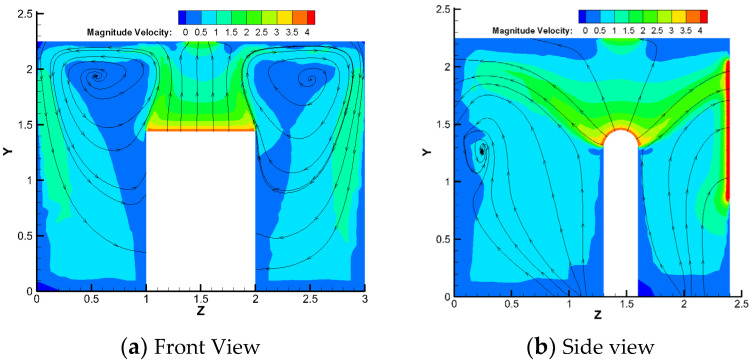
The velocity flow field inside the machine.

To clarify the flow field inside the machine shop, this section takes working condition 5 (local exhaust air volume of 600 m^3^/h (velocity of 2.36 m/s), particle emitting initial velocity of 3.93 m/s) as an example for the analysis of the positive and lateral velocity inside the machine shop, as shown in Figure 7.

Large vortices were formed at the left, right, and rear of the particle emission source, leading to an increase in the particle concentration. The velocity is higher at the slit of the loading door of the machine tool, causing many oil mist particles to escape. The wind speed at the local exhaust port decays too quickly, which has a certain attractive effect on the particles in the area directly below the source, reducing the efficiency of the local exhaust system for eliminating oil mist particles. The wind speed of the upper side of the dispersing source and the front and rear diffusion parts were approximately 2.5 m/s, and the vortex areas on the left and right sides were in the range of 0.5–1 m/s.

**Figure 8:**
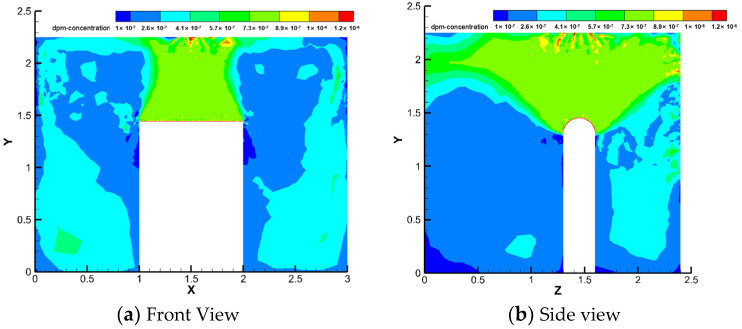
Particle concentration field inside the machine.

Under the same working conditions, this study analysed the particle concentration fields in the frontal and side views inside the machine tool, as shown in Figure 8.

Oil mist particles mainly gather in the emitting source directly above, and a large amount of diffusion to the front and back sides increases the concentration on both sides of the machine tool. The oil mist particles can diffuse outwards through the middle and upper gaps. The local exhaust is directly below the particle area concentration, and this part of the particles can be excluded by the local exhaust system. The concentration of particles on the upper side of the source and the front and rear diffusion parts is approximately 9.4 × 10^−7^ kg/m^3^ and that in the diffusion area on the left and right sides is 3.8 × 10^−7^ kg/m^3^.

## 3. Theoretical Analyses of the Purification and Separation Technology of Oil Mist Particles

The above analysis indicates that the escape of oil mist particles can be attributed to two scenarios. First, the capture efficiency of the local exhaust of oil mist particles in the closed machine tool was low, causing the particles to spread around the area. Second, the particle emitting an initial velocity is larger, leading to the escape of particles to the outside. By analysing the particle concentration field inside the confined machine, it was found that the left and right sides of the machine led to a larger particle concentration field owing to vortex entrainment. Therefore, this study attempted to increase the gas–water–oil mist separation device with a pressure-relief effect on the upper left and right sides of the machine according to the flow field characteristics of the machine tool. In this manner, the airflow direction can be controlled, the particles entrained in the airflow can be separated and removed, the pressure at the gap of the machine tool can be weakened, and particle escape can be reduced.

### 3.1. Mathematical Model of the Gas–Water–Oil Mist Separation Process

**Figure 9:**
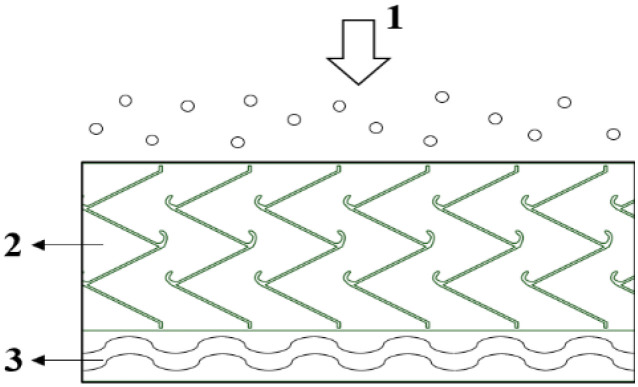
Principal diagram of the gas–oil separation device. 1—Airflow entry direction; 2—Inertial collision stage; 3—Filtration stage.

The gas–water–oil mist separation device was borrowed from the marine turbine inlet air–salt mist separation window [44]. Its fundamental principle is vapour–liquid separation technology, which is mainly used to remove tiny liquid droplets and impurities entrained in the airflow. It also plays the role of pressure relief so that the airflow in the machine changes direction and carries oil mist particles to the device. A schematic of the separation device is shown in Figure 9. When the airflow carrying oil mist particles enters the device, it first passes through the inertial collision stage, whose “W”-type folding plate with fold hooks guides the flow direction of the airflow [45,46]. Consequently, the airflow collided with the folding plate wall several times to achieve high particle separation efficiency. After the primary separation of the airflow, it went directly into the secondary filtration stage. Glass-fibre wool and other filled filter media layers intercept the small particle size particles, further enhancing the separation efficiency.

#### 3.1.1. Theoretical Calculation of Collision Levels

The oil mist particles follow the airflow in the curve of the folded plate channel after the air–water–oil mist mixture enters the collision stage. The majority of particles in the inertia and centrifugal effect of the wall of the folded plate are captured, and a small number of small particles follow the airflow to escape. The oil mist particles trapped on the wall of the folding plate gradually converge and deposit, thereby forming a layer of oil–water mixed film. When the airflow speed in the channel is extreme, it will make the liquid film break, regenerating small particles to follow the airflow to escape, thereby reducing the separation efficiency of the folding plate. The abovementioned situation is the more important secondary carryover problem in the folding plate. This study uses a droplet motion model to analyse the secondary carryover phenomenon generated by the shear effect of the liquid film in the airflow and to investigate the critical inlet velocity of the collision stage and the optimal structural parameters.

##### Causes of Liquid Film Rupture at the Wall of the Folding Plate

The factors that cause the avoidance of liquid film rupture include the velocity of the airflow, the materiality of the particles forming the liquid film, the thickness of the liquid film, and the structure of the folding plate. Certain assumptions are made to simplify the study, which are as follows: gravity is perpendicular to the direction of the airflow, so it is ignored; no separation occurs between the shear-generating airflow and the liquid film; the liquid film formed on the wall of the folding plate is extremely thin, where its internal flow can be considered a boundary layer flow [47].

**Figure 10:**
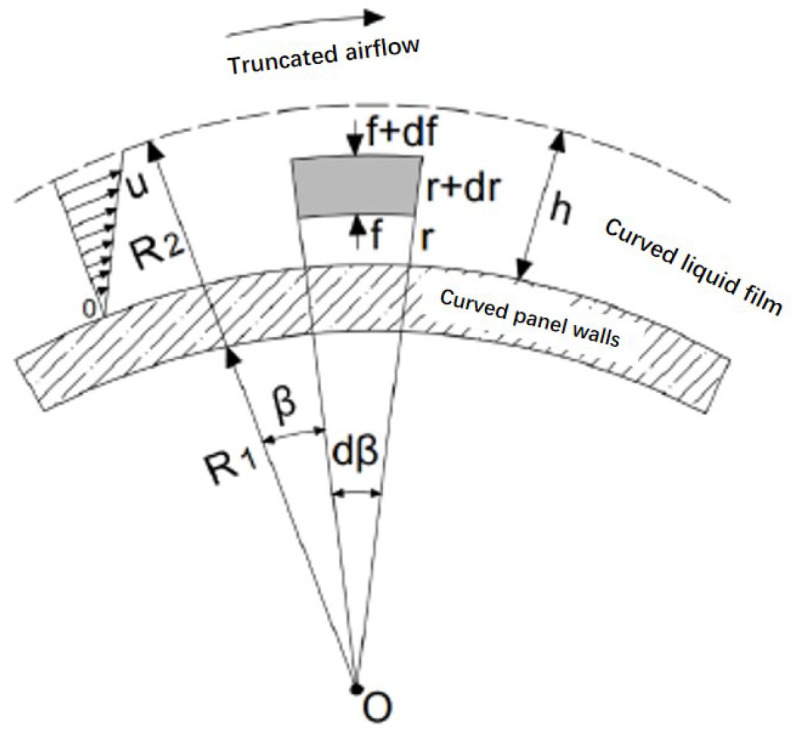
Force analysis of liquid film micro-element forces [47].

Figure 10 shows the force analysis of the liquid film microelement under the action of the shear flow. Its liquid film microelement in the r direction of the force includes centrifugal force and the pressure of the fluid above it, and its combined force can be expressed as Equation (8):

(8) $$\mathrm{dF}=\rho_{l}\cdot r\cdot d\beta \cdot \mathrm{dr}\cdot \frac{u^{2}}{r}+f\cdot r\cdot d\beta -\left(f+\mathrm{df}\right)\cdot \left(r+\mathrm{dr}\right)\cdot d\beta$$

where f—Centrifugal forces on fluid microelements, N/m^2^; β—Clamping angles corresponding to fluid microelements, °; u—Flow rate of the liquid film, m/s.

Integrating Equation (8) in the direction of liquid film thickness and ignoring the smaller term, Equation (8) is converted to Equation (9):

(9) $$F=d\beta \left[\int_{R_{1}}^{R_{2}}\rho_{l}\cdot u^{2}\cdot \mathrm{dr}-f\cdot \frac{1}{2}\int_{R_{1}}^{R_{2}}r^{2}\right],$$

The first term in the brackets to the right of Equation (9) is the centrifugal force term, and the second term is the pressure of the fluid above on the microelement or the adhesion of the fluid below to the microelement. The velocity distribution of the water film at the wall is calculated [48] as Equation (10):

(10) $$u=\frac{U}{h}y\;\left(y\in \left[0,h\right]\right),$$

where U—Flow rate of liquid on the surface of the liquid film, m/s; h—Thickness of the liquid film, m.

Substituting Equation (10) into Equation (9) and integrating again yield Equation (11):

(11) $$f\cdot r│\begin{array}{c} R_{2}\\ R_{1} \end{array}=f\left(R_{2}\right)R_{2}-f\left(R_{1}\right)R_{1}=\frac{\sigma }{R_{2}}R_{2}-f\left(R_{1}\right)R_{1},$$

where σ—Liquid film surface tension, N/m.

When the liquid film at the wall of the folding plate is not broken, the adhesion force at the inner boundary of the liquid film is greater than the surface tension of the liquid at the outer boundary. When the liquid film ruptures, the centrifugal force increases because of the increasing velocity of the airflow, which exceeds the surface tension of the liquid film, and rupture occurs. The forces in equilibrium are shown in Equation (12):

(12) $$\frac{\rho_{l}h}{3}U^{2}-\sigma =0,$$

where $\rho_{l}$ is the density of the liquid film, kg/m^3^.

From the horizontal direction, the shear stress of the airflow on the surface of the liquid film should be equal to the shear stress of the liquid film on the face of the folded plate wall, as shown in Equation (13):

(13) $$\tau_{g}=\tau_{w},$$

where $\tau_{g}$ is the shear stress on the liquid film surface by air flow, N/m^2^; $\tau_{w}$ is the shear stress on the liquid film at the wall of the folding plate, N/m^2^.

On the basis of the assumption of a thin liquid film [47,48], the shear stress on the liquid film at the wall of the folded plate and the shear stress on the liquid film from the airflow can be deduced as Equations (14) and (15), respectively:

(14) $$\tau_{w}=\mu_{l}\frac{\mathrm{du}}{\mathrm{dy}}{│}_{y=0}=\mu_{l}\frac{U}{h},$$

(15) $$\tau_{g}\approx \tau_{i}=\frac{1}{2}\rho_{g}C_{f}u_{g}{}^{2},$$

where $\mu_{l}\;$is the dynamic viscosity coefficient of the fluid film, Pa/s; $\rho_{g}$ is the density of the airflow, kg/m^3^; $C_{f}$ is the dimensionless resistance factor. Calculations can be made by using Equation (16):

(16) Cf=1.328Reg, Reg=ρguglμg (Reg<0.5−1×106),

where l is the folding plate spacing, m; $u_{g}$ is the airflow rate between folded panels, m/s; $\mu_{g}$ is the dynamic viscosity coefficient of the airflow, Pa/s.

From the joint derivation of Equations (12)–(16), the critical airflow velocity for liquid film fragmentation leading to secondary carryover is obtained, as shown in Equation (17):

(17) $$u_{g}=1.895{\left(\frac{{l\mu }_{l}{}^{2}\sigma }{\rho_{l}\rho_{g}\mu_{g}}\right)}^{1/3}\frac{1}{h},$$

##### Critical Airflow Inlet Velocity

When the pressure relief airflow enters the collision stage of the gas–water–oil mist separation device, the oil mist particles may form a thin liquid film on the wall of the plate owing to a collision with the folding plate. However, the thin liquid film will break because of the airflow speed between the plates, liquid film materiality, liquid film thickness, folding plate structure, and other factors. This results in secondary carryover and reduced separation efficiency.

(18) $$u_{g}=1.895\times {\left(\frac{l\times \mu_{l}{}^{2}\times \sigma }{\rho_{l}\times \rho_{g}\times \mu_{g}}\right)}^{1/3}\times \frac{1}{h},$$

where $u_{g}$ is the air flow rate between folded plates (m/s), l is the folding board spacing (m), $\mu_{g\;}$is the dynamic viscosity coefficient of airflow (Pa/s), $\rho_{g}\;$is the density of airflow (kg/m^3^), $\rho_{l}\;$is the density of liquid film (kg/m^3^), $\mu_{l}\;$is the fluid film dynamic viscosity coefficient (Pa/s), h is the thickness of liquid film (m), and σ is the liquid film surface tension (N/m).

**Figure 11:**
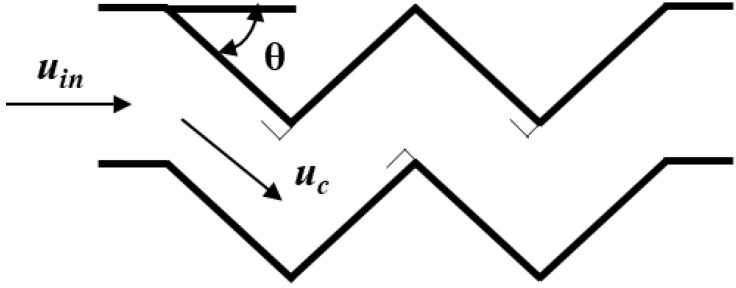
Relationship between airflow velocity and inlet velocity in the folded plate.

To facilitate the understanding and research, the airflow velocity between the folding plates is converted into the airflow inlet velocity, and the relationship between the two is shown in Figure 11 and Equation (19).

(19) $$u_{\mathrm{in}}=\cos\theta \times u_{g},$$

Substituting Equation (19) into Equation (18), the critical inlet velocity of the pressure-relief airflow can be obtained as

(20) $$u_{\mathrm{in}}=1.895\times {\left(\frac{l\times \cos^{3}\theta \times \mu_{l}{}^{2}\times \sigma }{\rho_{l}\times \rho_{g}\times \mu_{g}}\right)}^{1/3}\times \frac{1}{h},$$

where $u_{\mathrm{in}}$ denoted the critical speed of the collision level folding plate import (m/s), and θ is the collision level folding plate clamping angle (°).

#### 3.1.2. Structural Parameters

By analysing the inlet critical velocity (Equation (20)), it was found that the factors affecting the critical velocity mainly include the folding plate structure and the physical parameters of the liquid film and airflow.

**Figure 12:**
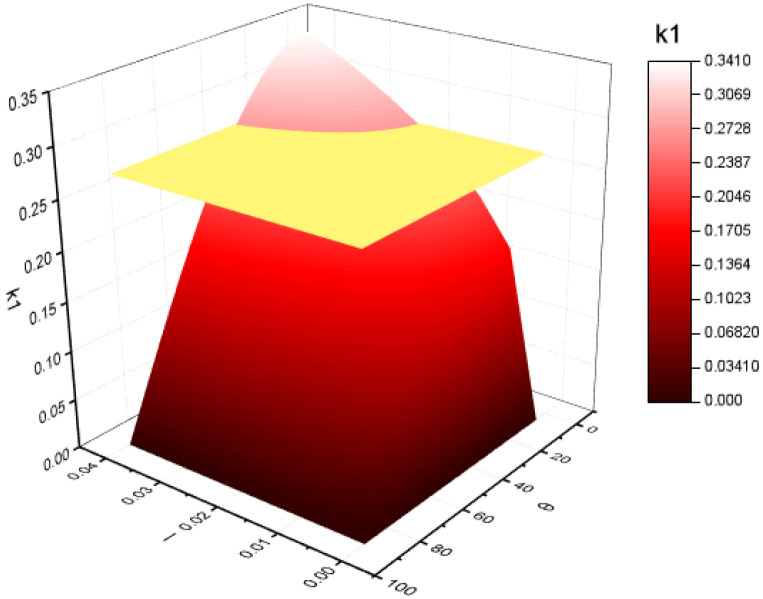
Variation of different plate spacing, folded plate angle, and k_1_ value.

Therefore, k_1_ is set as the folded plate structure coefficient, and k_2_ is the fluid physical parameter coefficient [47]. The expressions are as follows:

(21) $$k_{1}={\left(l\times \cos^{3}\theta \right)}^{1/3}={\left(d\times \cos^{3}\theta \right)}^{1/3},$$

(22) $$k_{2}={\left(\frac{\mu_{l}{}^{2}\times \sigma }{\rho_{l}\times \rho_{g}\times \mu_{g}}\right)}^{1/3},$$

k_1_ is the focus of this study since the fluid physical parameters required for k_2_ calculation are constant. The folding plate angle was set in the range of 0–90°, the plate spacing was set in the range of 0–40 mm [49], and k_1_ was calculated and plotted using Equation (21), as shown in Figure 12.

As shown in Figure 12, the maximum value of k_1_ is 0.32. Under the premise that the structure of the folding plate is reasonable and the pressure drop is small, more than 80% of the maximum value of k_1_ in the figure is selected as the ideal structural parameter to increase the critical velocity of the inlet of the folding plate as much as possible. The ideal range of the angle of the folding plate is 20–60°, and the ideal range of the plate spacing is 20–35 mm. In accordance with the literature, the plate spacing and plate angle of the collision stage have a greater influence on the separation efficiency and pressure loss. A large plate angle and a small plate spacing have a high separation efficiency, but the pressure loss increases. Thus, this section takes a comprehensive consideration that the collision stage is ideal when the plate angle is about 60° and the plate spacing is about 30 mm.

### 3.2. Numerical Simulation Using the Gas–Water Oil Mist Separation Device

#### 3.2.1. Physical Model

The physical model of the separation device mainly consisted of two parts: the inertial collision stage and the filter stage, 1.6 m long and 0.86 m wide. The W-shaped folding plate with a folding hook has a width of 0.66 m. The width of the folding plate accounts for 0.6 m, and the thickness of the plate is 0.001 m. The filter stage is 0.2 m wide.

**Figure 13:**
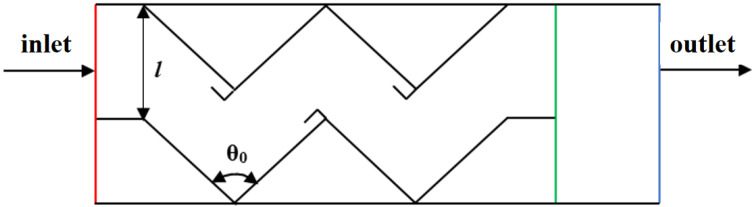
Schematic diagram of the local structural parameters of the model air-water-oil-mist separation unit.

In this study, we assume that the vertical distance of the folding plate of the inertial collision stage is l, the angle of the folding plate is $\theta_{0}$, the airflow enters from the left side of the folding plate and leaves from the right side of the filter stage, and the model structure parameters are schematically shown in Figure 13.

The boundary conditions considering the boundary types are set, as shown in Table 4.

#### 3.2.2. Working Conditions

In separation devices, the separation efficiency of the inertial collision stage depends mainly on the inertial separation, which is influenced by the airflow velocity, droplet particle size, folding plate angle, and folding plate spacing [37]. Therefore, the average flow velocity near the pressure relief port under the pressure relief strategy was measured to be 2.36 m/s. Therefore, 0.71 and 3.99 m/s were selected as the representative data and set as the inlet initial velocity of the device and the corresponding particle mass flow rate at this time. The parameter range of the folding plate angle and the folding plate spacing is considered, and the simulated working conditions of the gas–water oil mist separation device are set, as shown in Table 5.

## 4. Simulation Results and Discussion

### 4.1. Flow Field Analysis

**Figure 14:**
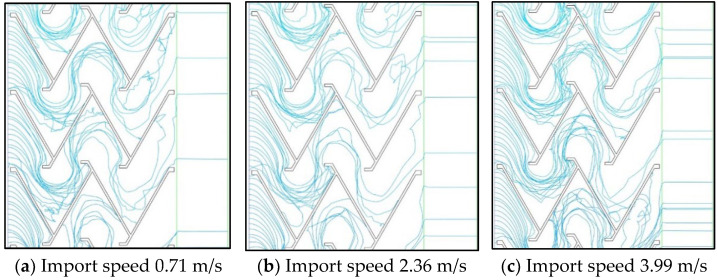
Particle motion trajectory.

**Figure 15:**
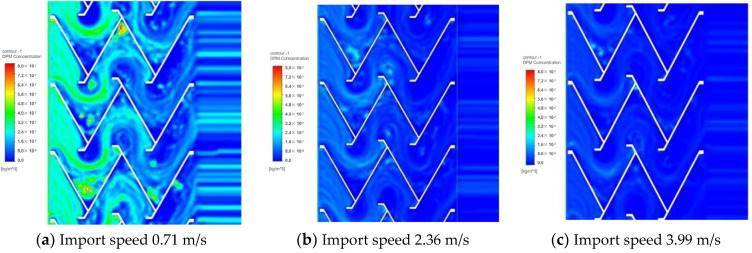
Particle concentration field.

The simulation results of the best folding plate structure with 60° folding plate angle and 30 mm folding plate spacing were selected to analyse the particle trajectory and particle concentration field of the gas–water–oil mist separation device, as shown in Figure 14 and Figure 15.

As shown in Figure 14, in the inertial collision stage, more particles hit the folding plate or folding hook in the first and second turns and are thus trapped by it. The particles with airflow in the third turn mostly flowed out of the region along the direction of the folding plate. When the speed is low, the probability of the particles hitting the folding plate in the three turns of the folding plate area is low, and the number of particles entering the filter area is high. When the speed increased, most of the particles in the first two turns of the folding plate area were captured by the folding plate, and the number of particles that collided with the folding plate at the later stage decreased.

Figure 15 shows that the particle concentration inside the model decreases significantly with an increase in the airflow inlet velocity. A substantial decrease in the particle concentration was also noted after passing through the inertial collision stage. The first V-shaped folding plate had a greater ability to capture particles, and the concentration of particles near the upper half of the V-shaped folding plate was larger. The lower part of the second V-shaped left folding plate had a certain probability of capturing particles, and its concentration was higher. The W-shaped folding plate had many particles trapped at the corners of the convergence, thus increasing the concentration in this region.

### 4.2. Effect of Airflow Inlet Velocity on Particle Separation Efficiency

**Figure 16:**
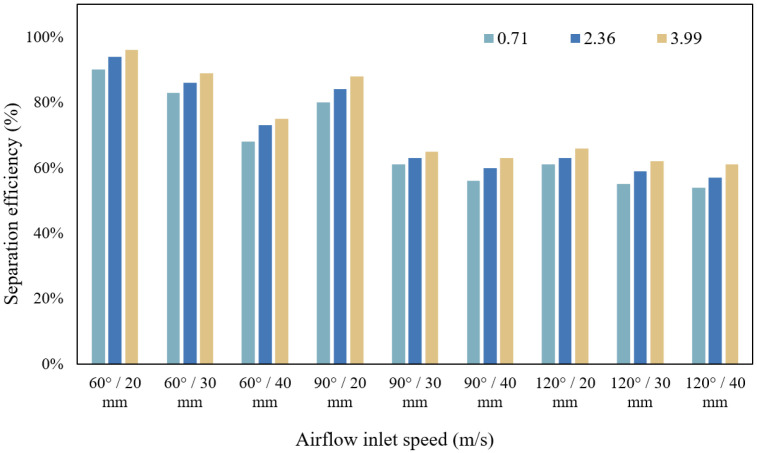
Relationship between gas inlet flow rate and separation efficiency.

The separation efficiency of different inlet flow rates when the angle of the folding plate is 60–120° and the distance between the folding plates is 20–40 mm is analysed, and the calculation results are plotted in Figure 16.

Figure 16 illustrates that the separation effect of the gas–water–oil mist separation device on the particles improves with the increase in airflow inlet velocity. When the airflow inlet speed was 3.99 m/s, the average separation efficiency was 74%. The higher the inlet speed, the shorter the time for the particles to collide with the folding plate, the lower the deflection force applied to the particles, and the better the separation efficiency. For the structural parameters of 60° and 20 mm, 60° and 30 mm, and 90° and 20 mm, the separation efficiency was above 80%, and the average separation efficiency was approximately 88%. This also indicates that the ideal structural parameters for gas–water–oil mist separation devices are more important.

### 4.3. Effect of Folding Plate Spacing on Particle Separation Efficiency

**Figure 17:**
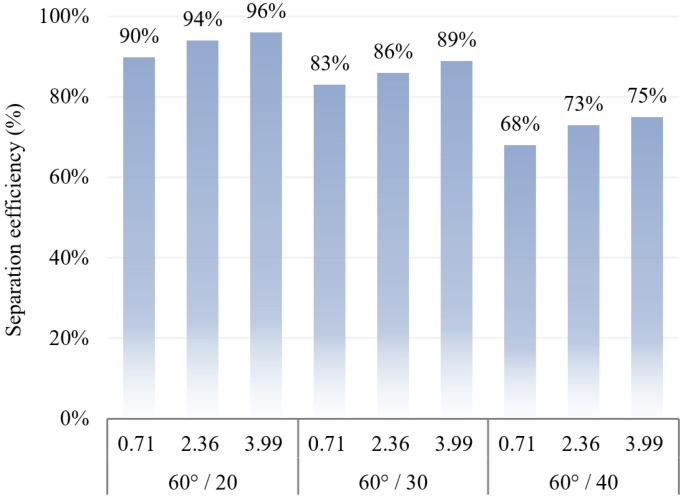
Relationship between folding plate spacing and separation efficiency.

The angle of the folding plate was set to 60°, and its effect on the separation efficiency was analysed by changing the folding plate spacing, as shown in Figure 17.

With an increase in the distance between the folding plates, the efficiency of the gas–water–oil mist separation device decreased by approximately 20%. At the same inlet speed, the separation device efficiency decreased by approximately 3%. This is because when the plate spacing decreases, the pushing force of the airflow in the device increases; thus, the probability of particles colliding with the plate wall increases, and the separation efficiency is improved.

### 4.4. Effect of Folding Plate Angle on Particle Separation Efficiency

**Figure 18:**
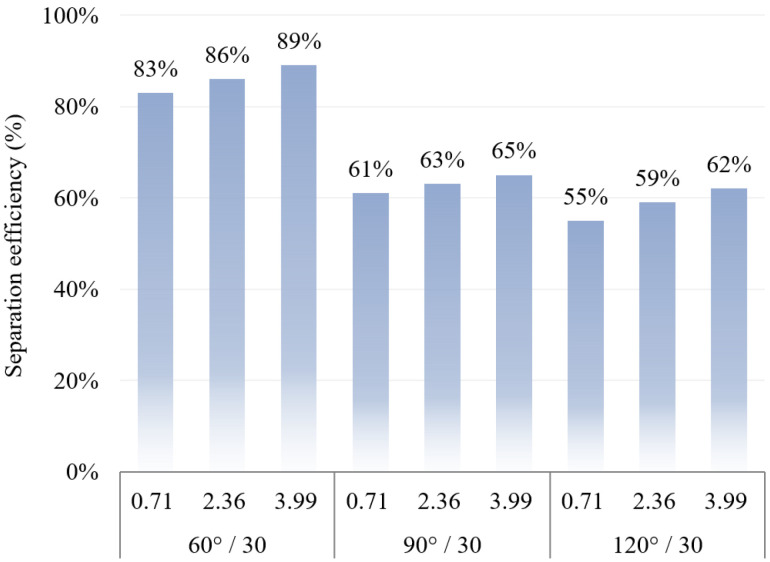
Relationship between the angle of the folding plate and separation efficiency.

The simulation results with a folded plate spacing of 30 mm were selected to analyse the separation efficiency under different folded plate angles, as shown in Figure 18.

A comparison of the separation efficiency at different angles is shown in Figure 18. The separation efficiency decreases remarkably when the separator angle increases from 60° to 90°, whereas the separation efficiency decreases slightly when the angle increases from 90° to 120°. The difference in separation efficiency between 60° and 120° folding plates at the same plate spacing is approximately 30%, indicating that the angle of the folding plate has a greater influence on the separation efficiency. This finding is mainly because the particle will accelerate until the frictional forces equal the centrifugal forces as the particle is deflected by centrifugal forces, where the net force on the particle is zero and moves at maximum speed. Therefore, the smaller angle of the folding plate indicates that the angle of deflection of the particles increases, resulting in an increase in the centrifugal force of the particles and in the separation efficiency of the device.

### 4.5. Control Effect after Adding Gas–Water–Oil Mist Separation Device

#### 4.5.1. Effect of Separation Device on Velocity Field and Particle Concentration Field

**Figure 19:**
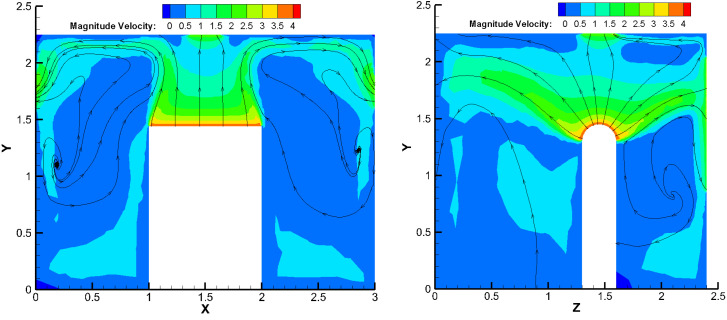
Velocity flow field inside the machine.

**Figure 20:**
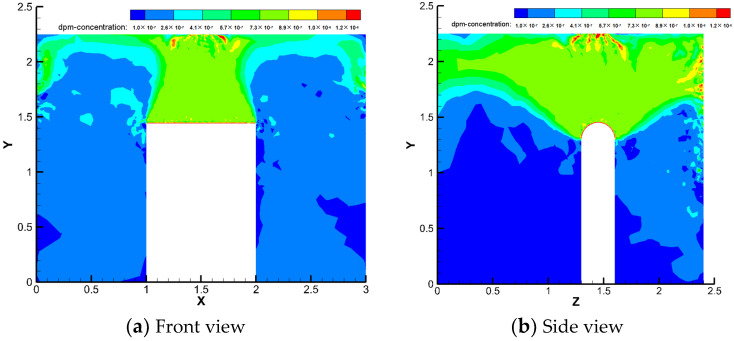
Internal particle concentration field distribution.

The simulated working conditions were set to be consistent with the configuration shown in Figure 7. The airflow and overall concentration field distributions within the machine after adding the air–water–oil mist separation device are shown in Figure 19 and Figure 20, respectively.

After adding the separation device on both sides of the machine, the cyclonic flow inside the machine on the left and right sides and rear was weakened. The flow field speed at the gap of the machine-loading door was also reduced. In addition, the gaps on the left and right sides tend to allow the flow back inside, thus weakening the flow of particles outwards. The higher concentration of oil mist in the machine continues to be concentrated in the middle and upper parts of the machine and on two sides. The change in airflow above the machine is more uniform, with the wind speed on the upper side of the emitting source and in the front and rear diffusion parts dropping from 2.5 m/s to approximately 1.5 m/s, and the average wind speed at the pressure relief being approximately 2.36 m/s.

**Figure 21:**
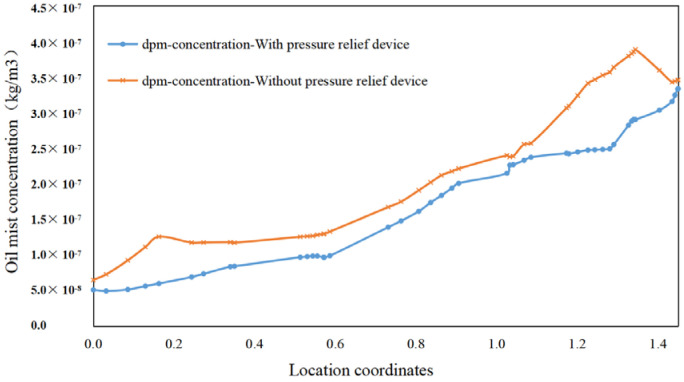
Difference in concentration with and without pressure relief device at L1.

**Figure 22:**
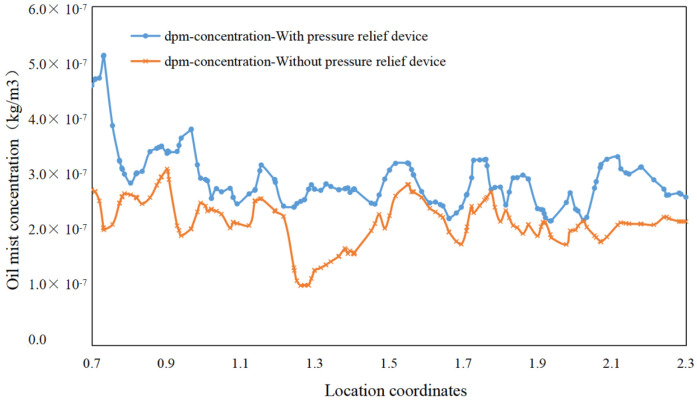
Difference in concentration with and without pressure relief device at L2.

Two lines near the pressure relief device, L1 (x = 0.3, y = 0–1.45, z = 0.5) and L2 (x = 0.2, y = 1.75, z = 0.7–2.3), were selected for variability analysis to observe more clearly the difference in the internal flow field of the air-port with and without the pressure relief device. As shown in Figure 21 and Figure 22 the concentration in the lower part of the machine decreases, whereas the concentration of oil mist at the pressure relief devices on two sides increases remarkably, and the airflow velocity increases.

This finding is because the pressure relief device drives the airflow on two sides and next to the gap in the loading door toward the pressure relief port due to the reduced turbulence of the internal flow field of the machine, resulting in a higher concentration of particles on two sides and a lower concentration of particles in the rest. This condition can cause a backflow of airflow at the gap, reducing the escape rate of particles at the gap in the machine. The particle concentration at the gap in the loading door of the machine decreases to 7.7 × 10^−7^ kg/m^3^, and the overall concentration in the space decreases and becomes progressively more homogeneous.

#### 4.5.2. Comparison of Pressure Values

**Figure 23:**
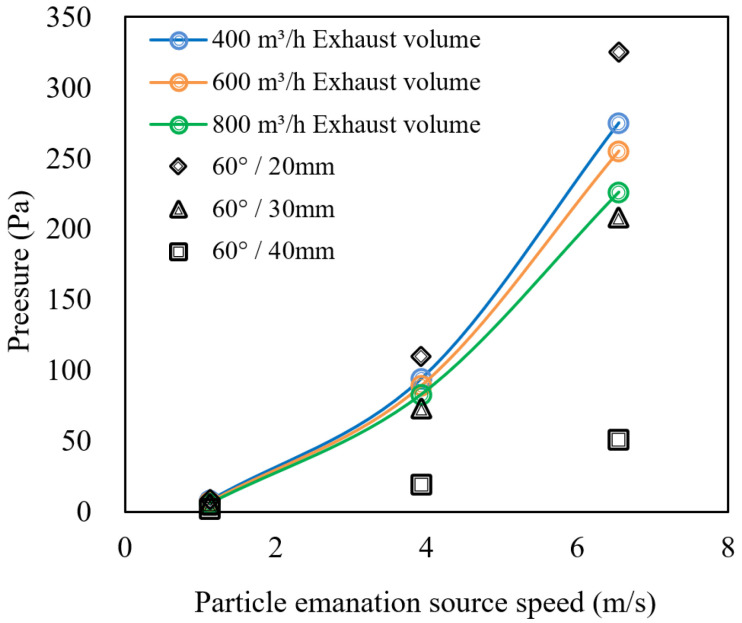
Pressure comparison of gas-oil separation device and closed machine model.

The applicability of the separation device in machine tools was analysed by comparing the internal pressure of the gas–water–oil mist separation device with that of the closed machine tool model. The simulation results are shown in Figure 23.

As shown in Figure 23, a particle dispersion velocity of 6.55 m/s has a greater effect on the internal pressure of the machine tool at different exhaust air volumes. For different folding plate spacings, the pressure loss of the gas–water–oil mist separation device at 20 mm was the largest, exceeding the internal pressure range of the machine tool. The pressure corresponding to each particle dispersion speed was 9–325 Pa. Moreover, the 30–40 mm plate spacing corresponding to each pressure value did not exceed the internal pressure range of the machine tool, and its pressure ranges were 5–208 Pa and 2–51 Pa. In accordance with the particle separation efficiencies calculated in Section 3.1.2 for different folding plate spacings, the air–water–oil–mist separation device with a folding plate spacing of 30 mm is the most suitable for the machine tool and is consistent with the theoretically calculated optimum folding plate structure parameters.

**Figure 24:**
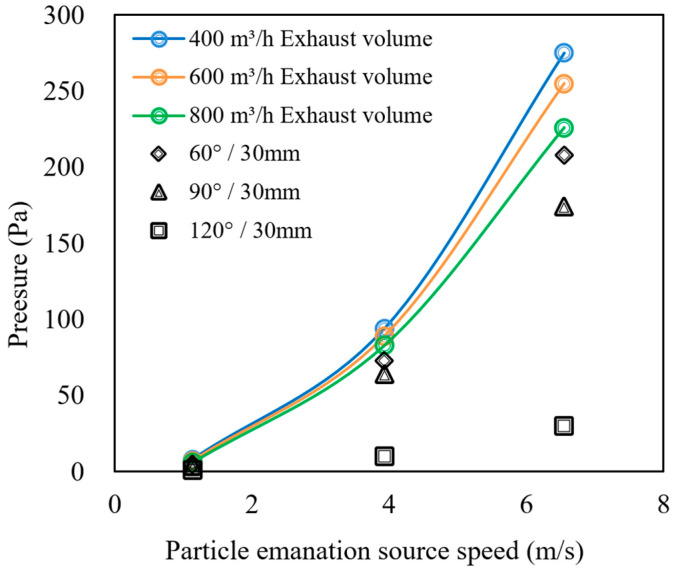
Pressure comparison of gas–oil separation device and closed machine model (2).

The simulation results obtained after the optimal folding plate spacing was derived are shown in Figure 24, which compares the internal pressure of the gas–oil separation device with the closed machine model at different folding plate angles.

Figure 24 shows that for different folding plate angles, the corresponding pressure values are lower than the internal pressure range of the machine. The pressure range does not vary significantly between 60° and 90°, with 5–208 Pa and 4–174 Pa, and the pressure range is the smallest at 120°, with 1–30 Pa. In accordance with the particle separation efficiencies calculated in Section 3.1.2 for different folding plate angles, a folding plate angle of 60° results in a more effective gas–water–oil–mist separation device, which is consistent with the theoretically calculated optimum folding plate structure parameters.

#### 4.5.3. Separation Efficiency of Oil Mist Particles

**Figure 25:**
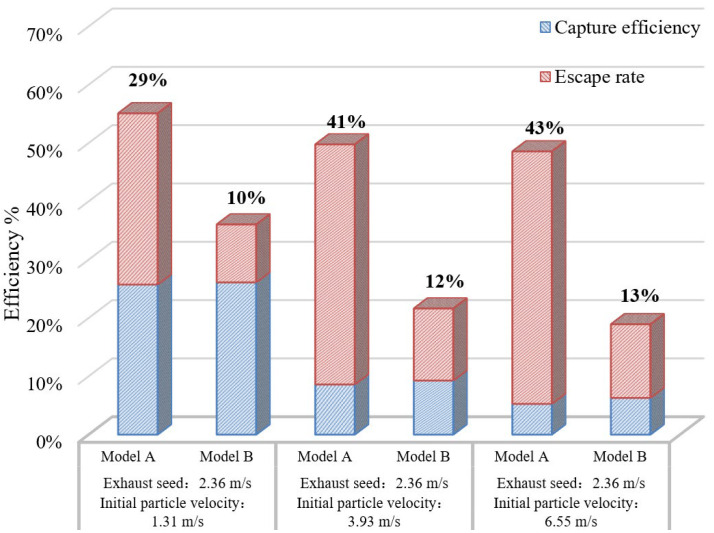
Comparative analysis of oil mist particle escape rate under the addition of gas–oil separation device.

The pressure relief airflow is subject to wind resistance when a gas–water–oil mist separation device is used at the pressure-relief port of the machine. Since the default pressure condition at the pressure-relief port has changed to the abovementioned pressure value, another numerical simulation is required. The abbreviated closed machine model refers to Model A, whereas the model with the new gas–water–oil mist separation device corresponds to Model B. A comparative analysis before and after the optimisation is shown in Figure 25. The escape rate of the oil mist particles can be obtained on this basis.

The overall escape rate of oil mist particles in Model B decreased significantly at different initial particle velocities, and the escape rate was approximately 10–13%. The reductions in the particle escape rate compared with Model A were 65.5%, 70.7%, and 69.8%, respectively. The escape rate was improved by approximately 5% compared to that when no additional pressure condition was provided at the pressure-relief port. This shows that the escape rate of the particles can be further reduced after the installation of the gas–water–oil mist separation device at the pressure-relief port.

## 5. Conclusions

At more than 50% of all sampling points in the workshop, the mass concentration of oil mist is above the standard value of 0.5 mg/m^3^. The gap in the loading door of the machine tends to create a large velocity exit, resulting in a large amount of entrained oil mist escaping and a high particle concentration of 9.4 × 10^7^ kg/m^3^. The airflow flows in an orderly manner toward the pressure relief opening, and the particle concentration at the gap of the machine loading door is reduced to 7.7 × 10^−7^ kg/m^3^ after the installation of additional gas–water–oil–mist separation devices on two sides of the machine, with a remarkable reduction in the escape rate, which is in the range of approximately 10–13%.

The theoretical derivation of the droplet motion shows that the factors influencing the critical velocity of the inlet of the gas–water–oil–mist separation device are divided into the structural factor k_1_ and the airflow materiality factor k_2_. From a practical point of view, the ideal structure of the folding plate section is calculated. The angle of the folding plate is approximately 60°, and the distance between the folding plates is approximately 30 mm. The separation efficiency of the device is above 80%, with an average separation efficiency of approximately 86%.

This study reveals the characteristics of oil mist particles emitted from the environmental zone of machine tool machining jobs and establishes a gas–water oil mist separation device to control the escape of oil mist particles to the outside while removing impurities, such as water vapor and oil mist particle aerosols. This device effectively realizes the “local control” of the pollution source, reduces the risk to personnel, increases the efficiency of exclusion, and has some practical reference value for improving the environmental pollution of oil mist particles in machining workshops.

## Figures and Tables

**Table 1:** Simulated working conditions.

Working Condition	Local Exhaust Air Volume (m^3^/h)	Local Exhaust Speed (m/s)	Initial Velocity of Particle Dispersion (m/s)		
1–3	400	1.57	1.31	3.93	6.55
4–6	600	2.36	1.31	3.93	6.55
7–9	800	3.14	1.31	3.93	6.55
10–11	1000	3.90	1.31	3.93	6.55

**Table 2:** CNC cutting machine three continuous measurement times data.

Number of Measurements	Concentration kg/m^3^	Dispersion Rate kg/s
Average of first measurement	5.41 × 10^−7^	7.160 × 10^−7^
Average of second measurement	5.44 × 10^−7^	7.184 × 10^−7^
Average of third measurement	5.28 × 10^−7^	7.156 × 10^−7^

**Table 3:** Comparison of measured data and simulated data.

No.	Dissemination Rate of Dispersal Sources (kg/s)	The Emission Rate of Machine Slits (kg/s)	Measured Emissivity (kg/s)	Relative Error %
1	1.0 × 10^−6^	6.51 × 10^−7^	7.17 × 10^−7^	9.2%
2	1.5 × 10^−6^	7.47 × 10^−7^	7.17 × 10^−7^	4.1%
3	2.0 × 10^−6^	9.63 × 10^−7^	7.17 × 10^−7^	25.58%

**Table 4:** Model boundary conditions.

Boundary	Types of Boundary Conditions	Temperature	DPM
Inlet	Velocity-inlet	300 K	escape
Outlet	Pressure-outlet		escape
Folded panels	Wall		trap
Internal boundaries	Interior		/
Upper and lower walls	Wall		trap

**Table 5:** Simulated working condition table.

Work Conditions	Import Speed (m/s)	Particle Mass Flow Rate (Kg/s)	Filtration Grade Porosity (%)	Folded Plate Spacing (mm)	Folded Plate Angle (°)		
1–9	0.71	3.01 × 10^−7^	55	20	60	90	120
				30	60	90	120
				40	60	90	120
10–18	2.36	3.88 × 10^−7^		20	60	90	120
				30	60	90	120
				40	60	90	120
19–27	3.99	4.01 × 10^−7^		20	60	90	120
				30	60	90	120
				40	60	90	120

## Data Availability

All data generated or analysed during this study are included in this published article.
